# Comparison of medial open wedge HTO in the treatment of medial unicompartmental knee osteoarthritis with mild and moderate varus

**DOI:** 10.3389/fsurg.2026.1942837

**Published:** 2026-10-02

**Authors:** Fuji Ren, Binyang Meng, Zi Zhang, Wenhe Li, Dong Zhao, Rui Wang, Jingmin Huang, Jiangang Cao

**Affiliations:** 1 Department of Sports Injury and Arthroscopy, Tianjin Hospital, Tianjin University, Tianjin, China; 2 Medical School of Tianjin University, Tianjin University, Tianjin, China

**Keywords:** articular cartilage, genu varus, joint-preserving surgery, knee, knee osteoarthritis, osteotomy

## Abstract

**Objective:**

This study aimed to evaluate the effectiveness of medial open wedge high tibial osteotomy (MOWHTO) in patients with medial unicompartmental knee osteoarthritis (OA) and mild to moderate varus deformity.

**Methods:**

A retrospective analysis was conducted on knee OA patients who underwent MOWHTO between January 2019 and January 2021, with a minimum follow-up of two years. Patients with medial unicompartmental knee osteoarthritis of Kellgren–Lawrence grade II–III were categorized by preoperative hip–knee–ankle (HKA) angle into mild varus (0° < HKA < 5°) and moderate varus (5° ≤ HKA < 10°) groups. The mild varus group included 30 patients (mean age, 57 ± 6 years), and the moderate varus group included 30 patients (mean age, 58 ± 5 years). Pre- and postoperative assessments included radiographic evaluations, arthroscopic findings, and clinical outcome measures.

**Results:**

Both groups demonstrated significant postoperative changes in HKA angle and weight-bearing line ratio, together with a significant increase in medial joint space width (*P* < 0.05). The distribution of Kellgren–Lawrence grades shifted toward lower grades at the 2-year assessment in both groups. Similarly, second-look arthroscopy at two years showed a shift toward lower International Cartilage Repair Society (ICRS) grades in both groups, with no statistically significant between-group differences (*P* > 0.05). Both groups demonstrated significant improvements in Short Form-12 (SF-12) and Knee Injury and Osteoarthritis Outcome Score (KOOS) measures (*P* < 0.05), while no statistically significant between-group differences were detected in postoperative KOOS or SF-12 scores (*P* > 0.05). Changes in KOOS and SF-12 scores exceeded the minimal clinically important difference (MCID) thresholds in both groups.

**Conclusion:**

MOWHTO was associated with favorable short-term outcomes in carefully selected patients with medial compartment knee osteoarthritis and mild or moderate varus deformity. No statistically significant differences in postoperative outcomes were detected between the two groups. Further comparative studies are required to determine the relative effectiveness of MOWHTO in patients with mild varus alignment.

## Introduction

High tibial osteotomy (HTO) is a well-established, knee-preserving surgical technique widely used to treat medial unicompartmental knee osteoarthritis (OA) or osteonecrosis associated with knee varus deformity, demonstrating favorable clinical outcomes (1–3). For cases with more severe varus deformity, lateral closing wedge HTO or double-level osteotomy may be necessary (3–6). However, the indication for HTO in patients with mild varus deformity remains debated, as these patients might, in some instances, benefit more from unicompartmental knee replacement (7). For younger, more active individuals, medial open wedge HTO may be a preferable option (7). While medial open wedge HTO has proven effective for treating early to mid-stage medial unicompartmental knee OA with significant varus deformity, the optimal approach for patients with mild varus deformity continues to be discussed among orthopedic surgeons (8). In such cases, medial open wedge HTO often requires minimal valgus correction, and whether these minor alignment changes can substantially improve clinical symptoms remains unclear.

Patient selection is crucial for achieving optimal outcomes in HTO (3), with the severity of varus deformity being a key consideration. Although varus deformity above a certain threshold often necessitates surgical correction, no universally accepted standard exists for determining this threshold. Generally, an HKA angle of ≥ 5° has been used as an indicator, although this criterion is somewhat arbitrary (9). Even patients without significant varus deformity may exhibit symptoms (10, 11). For instance, posterior root tears or complete radial tears of the medial meniscus may accelerate OA progression as the meniscus loses its stabilizing function (12). Furthermore, symptom severity does not always correlate with the degree of varus deformity. As such, determining whether to perform HTO based solely on the degree of varus deformity may not always be appropriate. Therefore, whether MOWHTO may be considered as a treatment option in carefully selected patients with medial compartment osteoarthritis and mild varus deformity warrants further investigation. This study assessed the clinical characteristics and postoperative outcomes of patients with medial unicompartmental knee OA and varying degrees of varus deformity who underwent HTO.

Although MOWHTO is well established for medial compartment osteoarthritis with more pronounced varus malalignment, evidence regarding its use in carefully selected patients with mild varus deformity remains limited. In particular, relatively few studies have simultaneously examined radiographic changes, arthroscopic cartilage appearance, and patient-reported outcomes in this population. Therefore, the present study aimed to evaluate the short-term outcomes of MOWHTO in patients with medial unicompartmental knee osteoarthritis and mild varus deformity (0° < HKA < 5°), using patients with moderate varus deformity (5° ≤ HKA < 10°) as a reference group. We hypothesized that carefully selected patients with mild varus deformity would demonstrate significant short-term improvement in clinical, radiographic, and arthroscopic outcomes after MOWHTO.

## Materials and methods

### General information

This retrospective study analyzed 148 patients who underwent medial open wedge HTO for knee osteoarthritis between January 2019 and January 2021. Of these, 88 cases were excluded for various reasons, including 20 patients with concomitant patellofemoral arthritis, 8 who underwent microfracture, 18 patients who underwent concomitant medial meniscus posterior root repair, 8 who underwent meniscal centralization, 6 with anterior cruciate ligament reconstruction, 6 who received contralateral HTO, and 7 with an HKA angle ≥ 10°. Additionally, 15 patients were excluded due to incomplete preoperative or follow-up data.

Ultimately, 60 patients were included in the study. These patients were categorized into two groups based on their preoperative HKA angle, measured from full-length weight-bearing anteroposterior x-rays of both lower limbs: the mild varus group (0° < HKA < 5°) and the moderate varus group (5° ≤ HKA < 10°) (13–15). The HKA angle was defined as the angle between the mechanical axis of the femur, which connects the center of the femoral head to the center of the knee joint, and the mechanical axis of the tibia, which connects the center of the knee joint to the center of the ankle joint (Figure 1). The center of the knee joint was defined as the midpoint of the line connecting the medial and lateral femoral condyles.

**Figure 1:**
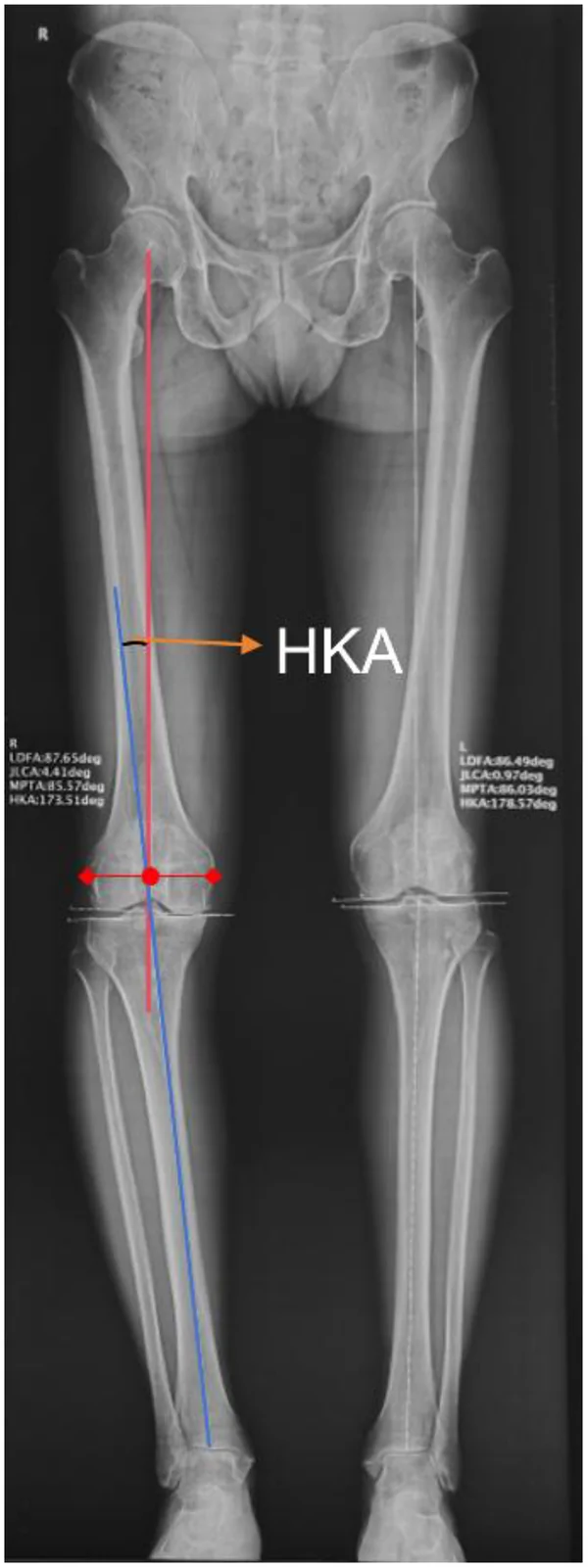
HKA: the acute angle formed by the intersection of the femoral mechanical axis (red line) and the tibial mechanical axis (blue line). The center of the knee joint was defined as the midpoint of the line connecting the medial and lateral femoral condyles.

All patients experienced medial knee pain and functional limitation despite prior conservative therapy. Varus deformity and medial joint tenderness were confirmed on examination. Standard radiographs and MRI were obtained preoperatively, and arthroscopic evaluation was performed at implant removal two years postoperatively. This study was approved by the Ethics Committee of Tianjin Hospital 2023189.

### Inclusion and exclusion criteria

#### Inclusion criteria

This study required patients to have a diagnosis of medial unicompartmental knee osteoarthritis with an HKA angle of less than 10° and to have undergone medial open wedge HTO treatment. Patients with medial unicompartmental knee OA of Kellgren-Lawrence (K-L) grade II–III were included. Patients were included if they had at least two years of postoperative follow-up data with complete and reliable records during this period. Additional inclusion criteria included having complete preoperative and postoperative imaging data, such as x-rays and MRI scans, and a preoperative knee cartilage condition assessed through arthroscopy or imaging, which excluded significant lateral compartment cartilage damage or other pathological conditions. Eligible patients were required to have a body mass index (BMI) within the range of 18.5 to 30 and no other knee joint diseases, such as gouty arthritis, rheumatoid arthritis, or knee joint infections. Furthermore, preoperative knee stability with no severe functional impairment and the ability to comply with postoperative rehabilitation protocols were essential.

#### Exclusion criteria

Exclusion criteria encompassed the presence of patellofemoral arthritis with a Kellgren-Lawrence (K-L) grade > III and concomitant procedures that could independently influence postoperative outcomes, including medial meniscus posterior root repair, meniscal centralization, microfracture, or anterior cruciate ligament reconstruction. The presence of a medial meniscus posterior root tear without concomitant root repair was not itself considered an exclusion criterion. Patients undergoing bilateral medial open-wedge HTO or those with incomplete or lost follow-up data were also excluded. Additional exclusion factors included the presence of systemic diseases such as severe diabetes or rheumatic diseases, a history of knee joint infection or infection-related complications, and a history of severe knee trauma, such as fractures or ligament tears, that could result in abnormal joint function. Patients with psychiatric conditions affecting postoperative recovery or follow-up compliance were also excluded, as were those who declined to participate in the study.

These concomitant procedures were excluded to reduce procedural heterogeneity and potential confounding. Patients undergoing bilateral HTO were also excluded to maintain independence of observations and to minimize the potential influence of bilateral rehabilitation on patient-reported outcomes.

### Surgical methodology

#### Preoperative planning

All procedures were performed by a team of two experienced orthopedic surgeons specialized in sports medicine and arthroscopy. Preoperative planning and correction angle calculations were based on the method described by Miniaci et al. (16). The primary objective was to achieve a target alignment line passing through the Fujisawa point (17), which is located at 62.5% of the total width of the tibial plateau, measured from the medial side.

#### Surgical indications

The indications for medial open wedge HTO included symptomatic medial unicompartmental knee OA, a K-L grade of ≥ 2, and the presence of varus deformity primarily originating from the extra-articular side of the tibia. Eligible patients were aged ≤ 65 years, with a flexion contracture < 15° and a knee flexion angle > 100°. Additionally, there were no significant degenerative changes in the patellofemoral joint or lateral compartment cartilage, and joint instability was absent.

Contraindications included a history of joint infection or inflammatory arthritis, symptomatic lateral compartment knee OA, significant sagittal plane knee instability, a flexion contracture > 15°, or a maximum knee flexion angle < 100°.

#### Surgical procedure

##### 1) Anesthesia and positioning

The surgical procedure began with anesthesia and positioning. Patients were placed in the supine position under either spinal-epidural combined or general anesthesia, and a tourniquet was applied for hemostasis. A barrier was placed at the level of the tourniquet on the operative side to facilitate opening the medial compartment during surgery. Fluoroscopy was used to mark the centers of the femoral head and ankle joint.

##### 2) Arthroscopic exploration and pathology management

Routine arthroscopic exploration was performed to assess cartilage and meniscal status. Meniscal tear morphology was recorded as posterior root, radial, horizontal, or complex tear. The presence of a medial meniscus posterior root tear itself was not an exclusion criterion; patients were excluded only when concomitant posterior root repair was performed. In the 11 patients with posterior root tears who remained in the study cohort, no root repair was performed, and all underwent limited arthroscopic debridement or partial meniscectomy according to the intraoperative findings. Unstable tissue associated with radial, horizontal, or complex meniscal tears was similarly treated with limited debridement or partial meniscectomy when clinically indicated. The osteotomy was canceled if significant degeneration or synovitis was identified in other compartments. Loose bodies were removed when present, and notchplasty was performed in cases of femoral notch impingement.

##### 3) Medial open wedge

For medial open-wedge HTO, a standard medial approach was used with a 7-cm incision. The pes anserinus was split, and the superficial medial collateral ligament was partially released as needed. Two parallel K-wires were placed approximately 3.5 cm below the joint line, directed toward the fibular head at an angle of about 8° to maintain the posterior tibial slope. A biplanar osteotomy was performed, preserving a 1 cm lateral cortical hinge. The opening gap was adjusted under fluoroscopic guidance to reach the target alignment through the Fujisawa point. Fixation was achieved using a T-shaped locked compression plate, and the osteotomy gap was filled with allograft cancellous bone.

#### Postoperative rehabilitation

Postoperative rehabilitation started once anesthesia wore off. Ankle pumps were initiated on the day of surgery, followed by quadriceps and straight-leg exercises on postoperative day one. Knee flexion training began on day two, aiming for full range by week four. Partial weight-bearing was allowed from day three, progressing to full weight-bearing by 4–6 weeks postoperatively.

#### Outcome measures

##### Follow-up and imaging evaluation

Follow-up evaluations were conducted at 6 weeks, 3 months, 6 months, 1 year, and 2 years postoperatively. Imaging assessments included anteroposterior and lateral x-rays of the knee, full-length weight-bearing anteroposterior x-rays of both lower limbs, Rosenberg view x-rays, and MRI scans, particularly at the 2-year follow-up.

##### Clinical outcome assessment

Clinical outcomes were assessed using the Knee Injury and Osteoarthritis Outcome Score (KOOS), which evaluates knee pain, symptoms, daily living activities, sports activities, and quality of life. Additionally, the Short Form-12 Health Survey (SF-12) was employed to measure health-related quality of life, focusing on both physical (Physical Component Score, PCS) and mental (Mental Component Score, MCS) components.

##### Arthroscopic evaluation

All 60 patients included in the final analysis underwent implant removal and second-look arthroscopy at approximately 2 years after MOWHTO. No patient included in the final cohort declined implant removal or second-look arthroscopy. During second-look arthroscopy, the macroscopic cartilage appearance of the medial femoral condyle and medial tibial plateau was evaluated using the International Cartilage Repair Society (ICRS) grading system. The postoperative arthroscopic assessments were based on the available operative records and arthroscopic images. Formal blinded reassessment and interobserver or intraobserver reliability analyses were not performed.

##### Imaging measurements

Imaging measurements were conducted using CARESTREAM VuePACS 11.1 software (Carestream Health, USA). Osteoarthritis severity was assessed according to the Kellgren–Lawrence (K-L) grading system using weight-bearing anteroposterior knee radiographs. Lower-limb alignment was evaluated using the HKA angle, weight-bearing line (WBL) ratio, and joint line convergence angle (JLCA). Additional measurements included medial joint space (MJS) width on Rosenberg-view radiographs, posterior tibial slope (PTS) on lateral knee radiographs, and medial meniscus extrusion (MME) and medial meniscus extrusion ratio (MMER) on MRI.

##### Arthroscopic cartilage assessment

The International Cartilage Repair Society (ICRS) grading system was used to assess the macroscopic arthroscopic appearance of the cartilage at the medial femoral condyle (MFC) and medial tibial plateau (MTP).

##### Clinical significance evaluation

The Minimal Clinically Important Difference (MCID) was applied to evaluate whether the observed improvements in KOOS and SF-12 scores reached clinical significance (18).

### Statistical analysis

Continuous variables were assessed for normality and are presented as mean ± standard deviation when normally distributed or as median (interquartile range) when non-normally distributed. For normally distributed continuous variables, preoperative and postoperative comparisons within groups were performed using paired-samples t-tests, whereas comparisons between the mild- and moderate-varus groups were performed using independent-samples t-tests. For non-normally distributed continuous variables, between-group comparisons were performed using the Mann–Whitney U test.

Categorical variables, including sex, Kellgren–Lawrence (K-L) grade, and International Cartilage Repair Society (ICRS) grade, were expressed as frequencies and percentages and analyzed using Pearson’s chi-square test. Fisher’s exact test was used when the assumptions of the chi-square test were not met.

No formal adjustment for multiple comparisons was applied. Because multiple radiographic, arthroscopic, and patient-reported outcomes were evaluated, the possibility of an increased Type I error rate should be considered when interpreting individual P values. Accordingly, these analyses were regarded as exploratory, and statistically significant findings were interpreted cautiously.

Because this was a retrospective study based on an available clinical cohort, no *a priori* sample size calculation was performed. Given the relatively small sample size of 30 patients per group, the possibility of insufficient statistical power and Type II error should be considered when interpreting nonsignificant between-group differences. Accordingly, the absence of a statistically significant difference was not interpreted as evidence of equivalence between groups.

All statistical analyses were performed using SPSS version 26.0 (IBM Corp., Armonk, NY, USA). All tests were two-sided, and a *P* value < 0.05 was considered statistically significant.

## Results

### Comparison of baseline data between the mild varus group and moderate varus group

There were no significant differences between the mild varus and moderate varus groups in terms of age, sex, affected side, body mass index (BMI), duration of symptoms, follow-up time, or range of knee flexion and extension (*P* > 0.05, Table 1).

**Table 1:** Comparison of baseline data between groups.

Baseline data		Mild Varus Group	Moderate Varus Group	*P*
Age		57 ± 5.61	58 ± 5.30	0.31
Sex(%)	male	11 (36.7)	12 (40.0)	0.79
	female	19 (63.3)	18 (60.0)	
Side(%)	right	13 (43.3)	14 (46.7)	0.80
	left	17 (56.7)	16 (53.3)	
BMI(Kg/m2)		26 ± 2.55	25.5 ± 3.02	0.45
Duration of symptoms(months)		11.5 ± 6.19	10.7 ± 5.80	0.64
Follow-up time(months)		26 ± 1.52	27 ± 2.32	0.40
Straightness-limited angle(°)		1.1 ± 1.69	1.4 ± 1.63	0.44
Maximum flexion angle(°)		133.9 ± 6.38	133.3 ± 4.96	0.67
Correction angle(°)		6.8 ± 0.69	10.9 ± 1.43	0.00
*Note: BMI, body mass index.*				

### General surgical outcomes

Routine arthroscopic exploration in all 60 cases did not reveal severe degeneration in other compartments. The bone union was achieved in all cases without instances of nonunion or delayed union at the osteotomy site (Table 2). Healing time was defined as the period from surgery until radiographic evidence of complete osteotomy gap union. (Radiographic union was defined as the presence of continuous bridging trabeculae across the entire osteotomy gap on both anteroposterior and lateral radiographs).

**Table 2:** Comparison of general outcomes of operation between groups.

Operation results		Mild Varus Group	Moderate Varus Group	*P*
Medial meniscal tear morphology (%)	Posterior root tear	5 (17%)	6 (20%)	0.94
	Radial tear	9 (30%)	9 (30%)	
	Horizontal tear	9 (30%)	7 (23%)	
	Complex tear	7 (23%)	8 (27%)	
Intercondylar fossa plasty (%)		14 (47%)	17 (57%)	0.61
Free body extraction (%)		6 (20%)	5 (17%)	1.00
Operative time (hours)		1.2 ± 0.27	1.2 ± 0.25	0.93
Intraoperative blood loss (mL)		69 ± 21.29	69 ± 20.44	0.95
Wound Drainage (mL)		110 ± 54.02	110 ± 45.81	0.97
Healing Time (months)		4.7 ± 1.18	4.9 ± 1.13	0.44

### Radiographic outcomes

Preoperatively, statistically significant differences were observed between the mild and moderate varus groups in both the HKA angle and WBL ratio (*P* < 0.05), with the moderate varus group exhibiting a larger HKA. Postoperatively, there were no statistically significant differences between the groups in HKA or WBL ratio (*P* > 0.05).

Preoperatively and postoperatively, no statistically significant differences were identified in PTS between the groups (*P* > 0.05). Similarly, MJS measurements revealed no significant differences between groups preoperatively or postoperatively (*P* > 0.05); however, both groups showed significant improvement in MJS postoperatively compared to preoperatively (*P* < 0.05, Table 3).

**Table 3:** Comparison of pre- and postoperative radiological findings between groups .

Parameter	Mild Varus Group		Moderate Varus Group		*P1*	*P2*	*P3*	*P4*
	pre-op(A)	2 years post-op(B)	pre-op(C)	2 years post-op(D)	A vs. B	C vs. D	A vs. C	B vs. D
HKA(°)	3.60 ± 0.64 (Varus)	3.80 ± 0.71 (Valgus)	7.87 ± 1.44 (Varus)	3.68 ± 0.86 (Valgus)	< 0.05	< 0.05	< 0.05	0.55
WBL(%)	45.98 ± 1.13	62.28 ± 2.81	29.37 ± 7.40	61.51 ± 1.90	< 0.05	< 0.05	< 0.05	0.22
JLCA(°)	2.96 ± 1.18	1.45 ± 0.56	2.54 ± 0.87	1.29 ± 0.52	< 0.05	< 0.05	0.12	0.26
PTS(°)	8.08 ± 0.74	8.44 ± 0.72	8.19 ± 0.79	8.57 ± 0.67	0.06	0.05	0.59	0.91
MJS(mm)	2.28 ± 1.01	3.16 ± 0.45	2.56 ± 1.15	3.38 ± 0.44	< 0.05	< 0.05	0.32	0.06
K-L(1/2/3/4)	0/12/18/0	0/22/8/0	0/13/17/0	0/21/9/0	< 0.05	< 0.05	0.94	1
MME (mm)	2.98 ± 1.19	3.06 ± 1.01	3.21 ± 0.67	3.36 ± 0.59	0.78	0.37	0.36	0.17
MMER (%)	35.32 ± 15.31	36.13 ± 13.60	34.25 ± 11.49	35.54 ± 12.19	0.83	0.68	0.76	0.86
MFC(ICRS 1/2/3/4)	0/14/7/9	2/22/5/1	0/10/11/9	1/24/4/1	< 0.05	< 0.05	0.50	0.90
MTC(ICRS 1/2/3/4)	0/3/19/8	2/16/10/2	0/5/16/9	1/18/8/3	< 0.05	< 0.05	0.77	0.84

HKA, hip-knee-ankle; WBL, weight-bearing line; JLCA, joint line converge angle; PTS, posterior tibial slope; MJS, medial joint space; KL, kellgren-lawrence; MME, medial meniscus extrusion; MMER, medial meniscus extrusion ratio; ICRS, international cartilage repair society; MFC, medial femur cartilage; MTC, medial tibial cartilage.

The distribution of K-L grades shifted toward lower grades at the 2-year postoperative assessment compared with baseline in both groups (*P* < 0.05). No statistically significant between-group differences in K-L grade distribution were detected either preoperatively or postoperatively (*P* > 0.05).

There were no statistically significant differences between the mild and moderate varus groups in MME or MMER preoperatively and postoperatively (*P* > 0.05). Furthermore, neither group showed significant improvement in MME or MMER postoperatively compared to preoperatively (*P* > 0.05, Table 3).

### Arthroscopic cartilage grading

There were no statistically significant differences in the ICRS grading of the FMC and tibial medial plateau cartilage between the mild varus and moderate varus groups, both preoperatively and postoperatively (*P* > 0.05, Table 3). At the 2-year postoperative assessment, the distribution of ICRS grades shifted toward lower grades in both groups compared with baseline (*P* < 0.05), while no statistically significant between-group differences were detected (*P* > 0.05; Figure 2).

**Figure 2:**
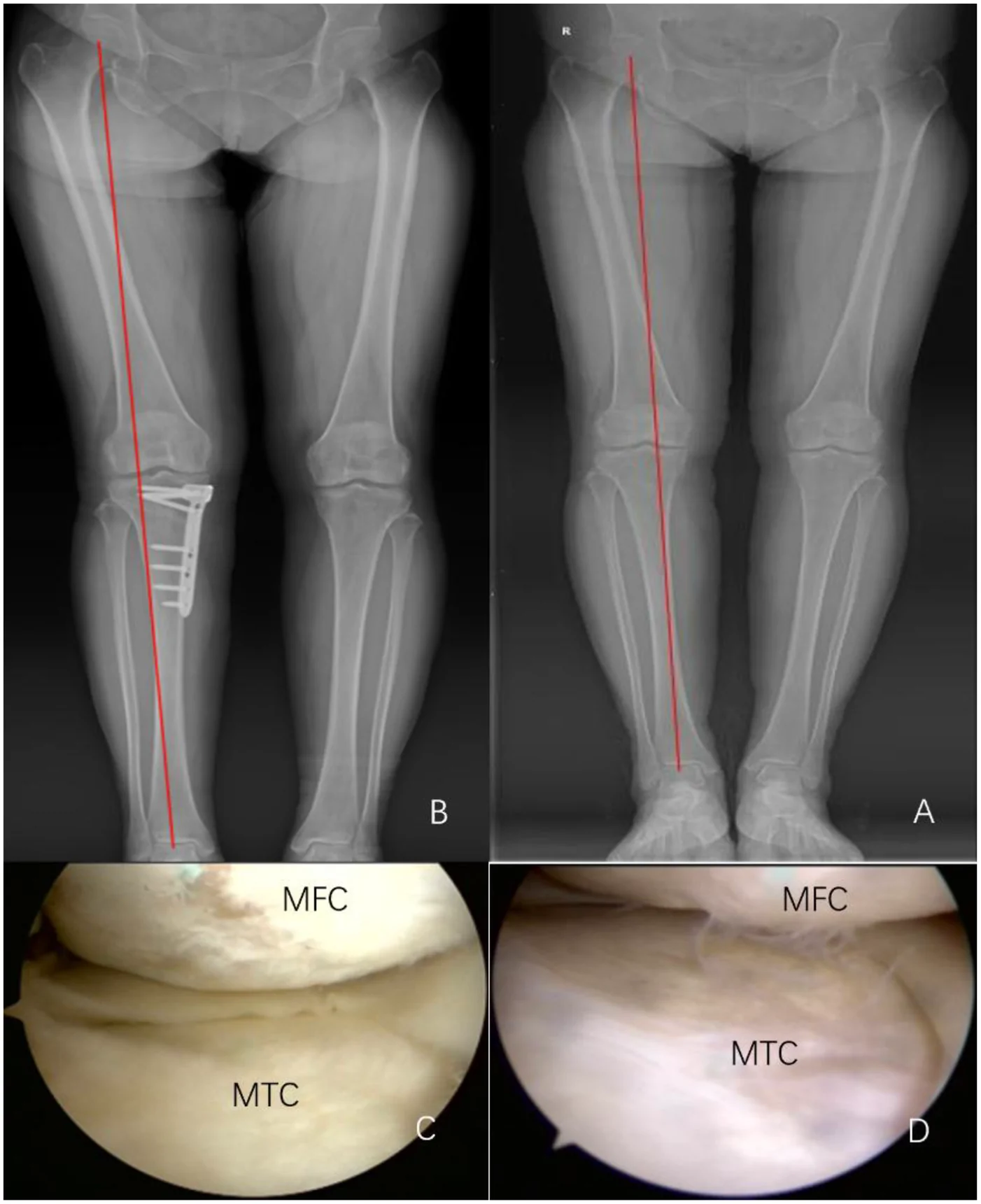
A 54-year-old female patient in the mild varus group. **(A)** Preoperative full-length weight-bearing anteroposterior radiograph showing varus lower-limb alignment. **(B)** Postoperative full-length weight-bearing anteroposterior radiograph after MOWHTO showing corrected lower-limb alignment and internal fixation. **(C)** Initial arthroscopic evaluation showing ICRS cartilage grading: MFC grade 4 and MTC grade 3 in the medial compartment. **(D)** Second-look arthroscopic evaluation at 2 years postoperatively showing an improved macroscopic cartilage surface appearance, with ICRS grade 2 at both the MFC and MTC.

### Patient-reported outcome measures (PROMs)

Both the mild and moderate varus groups showed significant postoperative improvements in Short Form-12 (SF-12) and KOOS subscale scores compared to preoperative scores (Table 4). No significant differences were found between the groups in either preoperative or postoperative SF-12 and KOOS scores (Table 4). Furthermore, changes in SF-12 and KOOS scores from preoperative to postoperative exceeded the minimum clinically important difference (MCID) thresholds in both groups, with no significant inter-group differences noted in these changes (Table 5).

**Table 4:** Comparison of pre- and postoperative PROMs between groups.

Outcome measure	Mild Varus Group		Moderate Varus Group		*P1*	*P2*	*P3*	*P4*
	pre-op(A)	2 years post-op(B)	pre-op(C)	2 years post-op(D)	A vs. B	C vs. D	A vs. C	B vs. D
SF-12								
Body composition	29.97 ± 5.41	50.80 ± 7.30	29.80 ± 5.33	51.36 ± 7.31	< 0.05	< 0.05	0.90	0.77
spiritual component	41.46 ± 6.11	55.30 ± 6.03	40.58 ± 6.27	55.86 ± 6.07	< 0.05	< 0.05	0.58	0.72
KOOS scores								
Pain	39.81 ± 11.33	90.55 ± 3.38	38.61 ± 10.78	91.10 ± 3.28	< 0.05	< 0.05	0.68	0.52
Symptoms	49.52 ± 16.77	85.71 ± 6.37	47.61 ± 16.97	87.62 ± 5.77	< 0.05	< 0.05	0.66	0.23
Daily life	48.13 ± 7.47	89.30 ± 5.09	47.45 ± 6.77	88.91 ± 4.25	< 0.05	< 0.05	0.71	0.75
Exercise	29.00 ± 16.47	76.67 ± 9.32	28.00 ± 16.06	75.67 ± 7.16	< 0.05	< 0.05	0.81	0.64
Quality of life	33.99 ± 18.26	76.27 ± 8.58	32.94 ± 17.06	75.23 ± 9.05	< 0.05	< 0.05	0.82	0.65

SF-12, Short Form-12; KOOS, Knee injury and osteoarthritis outcome.

**Table 5:** Comparison of changes in PROMs between groups.

Outcome measure	Mild Varus Group	Moderate Varus Group	*P*	MCID
SF-12				
*Δ* body composition	20.83 ± 10.66	21.56 ± 10.50	0.79	7.3
*Δ* spiritual component	13.84 ± 6.69	15.28 ± 6.33	0.39	6.3
KOOS				
*Δ* pain	50.74 ± 10.99	52.49 ± 10.09	0.52	15.4
*Δ* symptoms	36.19 ± 17.99	40.01 ± 17.69	0.41	15.1
*Δ* daily life	41.17 ± 7.24	41.46 ± 7.86	0.88	17.0
*Δ* movement	47.67 ± 21.49	47.67 ± 18.60	1.00	11.2
*Δ* quality of life	42.28 ± 18.04	42.29 ± 17.35	1.00	16.5

SF-12,Short Form-12;KOOS,Knee injury and osteoarthritis outcome;MCID,Minimal clinically important difference.

### Surgical complications

No intraoperative nerve or vascular injuries were reported, nor were there any cases of internal fixation failure, intra-articular fractures, or delayed or non-union of the osteotomy site. Postoperatively, seven cases of deep vein thrombosis were identified: three in the mild varus group and four in the moderate varus group. Of these, five involved venous thrombi in the intermuscular veins, one was a single thrombus in the peroneal vein, and one was a single thrombus in the posterior tibial vein(intermuscular vein thrombosis was diagnosed using Doppler ultrasonography). All patients were treated with oral rivaroxaban (20 mg daily) for 3 weeks to 3 months, and weight-bearing was delayed to 3–4 weeks postoperatively.

Two cases of Type I Takeuchi hinge point fractures occurred, one in each group (mild and moderate varus), along with one case of Type II Takeuchi hinge point fracture in the moderate varus group. These fractures were managed intraoperatively using compression plating, ensuring stabilization of the osteotomy sites. Weight-bearing was delayed to 3 weeks postoperatively for these cases.

At 2 months post-surgery, two patients (2/60) showed a maximum knee flexion of about 90°, but both gradually regained normal range of motion with rehabilitation therapy.

## Discussion

This study demonstrates that medial open-wedge HTO yields favorable short-term clinical outcomes for patients with medial unicompartmental knee osteoarthritis and mild (0° < HKA < 5°) or moderate (5° ≤ HKA < 10°) varus deformity. These findings align with those of Na et al. (19), who reported that even minimal correction of lower limb alignment can significantly improve clinical symptoms in patients with mild knee varus.

HTO is an established procedure for correcting varus deformity in medial knee osteoarthritis (1, 3, 5), though its indication for mild varus remains controversial (9), especially in younger patients unsuitable for unicompartmental arthroplasty. In this study, patients in both groups showed similar baseline characteristics, functional status, and symptoms, except for the preoperative HKA angle. Both groups demonstrated significant postoperative improvements in clinical outcomes following MOWHTO, and no statistically significant differences in postoperative PROMs were detected between the mild- and moderate-varus groups. These findings suggest that favorable short-term outcomes may be achieved after MOWHTO in carefully selected patients with medial compartment osteoarthritis and mild varus alignment. However, because all patients in the present study underwent MOWHTO and no conservative-treatment or alternative-surgical-treatment control group was included, our findings should not be interpreted as demonstrating that MOWHTO is necessary or superior to other treatment strategies in patients with mild varus deformity. Treatment decisions should therefore remain individualized according to symptoms, age, activity level, meniscal status, cartilage condition, and overall lower-limb alignment.

Postoperative lower limb alignment accuracy is vital for the success of HTO. Previous studies suggest under-correction may affect outcomes, but our study did not directly compare such cases. This study highlights the importance of meticulous preoperative planning and intraoperative adjustments to achieve optimal alignment, consistent with the Fujisawa point. Although correction toward the Fujisawa point is widely used, the optimal degree of valgus alignment remains controversial, and individualized correction strategies are increasingly recommended. Care intraoperative corrections are crucial to avoid complications in cases with unique challenges, such as restricted knee extension or lateral discoid meniscus tears.

A postoperative shift toward lower K-L grades was observed in both groups; however, this finding should be interpreted cautiously. K-L grading incorporates radiographic joint-space narrowing, and MOWHTO substantially changes lower-limb alignment and load distribution across the medial compartment. In the present study, medial joint-space width increased after surgery in both groups. Therefore, the observed changes in K-L grade may partly reflect altered mechanical loading, joint-space appearance, and radiographic projection following alignment correction rather than true structural reversal of osteoarthritis. Furthermore, formal interobserver and intraobserver reliability assessments were not performed for K-L grading, which represents an additional methodological limitation. Accordingly, the postoperative shift in K-L grade should be regarded as a change in radiographic appearance rather than evidence of reversal of the underlying osteoarthritic process. A shift toward lower ICRS grades was also observed at second-look arthroscopy in both groups. However, these findings should be interpreted as changes in macroscopic arthroscopic cartilage appearance rather than evidence of true biological cartilage regeneration. Arthroscopy permits direct visualization of the cartilage surface but cannot determine the histological composition, biochemical properties, or mechanical quality of the observed tissue. In the absence of histological assessment, quantitative cartilage MRI, or other tissue-level validation, it cannot be determined whether the observed surface changes represent hyaline cartilage restoration, fibrocartilaginous repair, or other forms of tissue remodeling. Therefore, the arthroscopic findings in the present study support an improvement in cartilage surface appearance but do not establish true cartilage regeneration. In addition, the original arthroscopic assessments were not performed within a formal blinded reliability framework, and interobserver and intraobserver reliability were not evaluated; therefore, some degree of observer-related measurement variability cannot be excluded.

The use of adjunctive arthroscopy in HTO may impact short-term outcomes, but arthroscopy in our cohort did not alter surgical plans. Yun Changjun et al. (20) suggested that arthroscopy can provide additional short-term benefits, although these effects diminish over time. To ensure that the effects of HTO were evaluated independently, we excluded patients who underwent complex arthroscopic procedures.

This study has several limitations. First, as a retrospective study, it is inherently subject to selection bias and residual confounding. In particular, the decision to perform MOWHTO in patients with mild varus deformity was based on clinical judgment rather than random treatment allocation, introducing potential indication-related and surgeon-related selection bias. In addition, activity demands and patient preference were not systematically quantified as predefined study variables, although these factors may have influenced both the decision to undergo MOWHTO and postoperative outcomes. The final study cohort was also relatively highly selected. A substantial proportion of the initial MOWHTO population was excluded, particularly patients undergoing concomitant medial meniscus posterior root repair, meniscal centralization, microfracture, anterior cruciate ligament reconstruction, or bilateral HTO. These exclusions were intended to reduce the potential confounding effects of additional procedures and to improve the homogeneity of the study cohort. However, this approach may have reduced the external validity of our findings, because patients with medial compartment osteoarthritis and mild varus alignment frequently present with concomitant meniscal, cartilage, or ligamentous abnormalities in routine clinical practice. Therefore, the present findings should primarily be interpreted as applicable to carefully selected patients undergoing unilateral MOWHTO without major concomitant reconstructive or cartilage procedures and should not be generalized to the entire population of patients with mild varus osteoarthritis. Second, the sample size was limited to 30 patients per group, which may have reduced the statistical power of the study. Furthermore, all patients were recruited from a single institution and were of Asian descent, which may further limit the generalizability of the findings to other populations. In addition, K-L grading was based on retrospective radiographic assessment without formal interobserver or intraobserver reliability testing, and postoperative changes in limb alignment and radiographic joint-space appearance may have influenced the observed grading changes.

Moreover, the follow-up period of two years allows only short-term evaluation and may be insufficient to assess long-term osteoarthritis progression, osteotomy survival, conversion to arthroplasty, or the durability of the observed clinical and arthroscopic changes. Longer-term follow-up is therefore required. Although major concomitant reconstructive or cartilage procedures were excluded, minor intraoperative interventions, such as limited meniscal debridement or notchplasty, were permitted when clinically indicated. These procedures may have introduced residual treatment heterogeneity and could have influenced postoperative outcomes. In addition, the relatively small numbers within each meniscal tear subtype precluded adequately powered subgroup analyses, and residual confounding related to meniscal pathology cannot be excluded. Furthermore, although second-look arthroscopy allowed direct assessment of cartilage surface appearance, no histological evaluation or quantitative cartilage imaging was performed. Therefore, the observed changes in ICRS grade cannot establish the histological nature or biological regeneration of cartilage tissue. Moreover, the arthroscopic assessments were not supported by formal blinded reassessment or interobserver and intraobserver reliability testing, which may have introduced observer-related variability.

Furthermore, the absence of a comparison group receiving conservative treatment, isolated meniscal surgery, unicompartmental knee arthroplasty, or another treatment strategy prevents determination of whether MOWHTO is superior or preferable to alternative treatments in patients with mild varus alignment. Future prospective, multicenter studies with larger sample sizes, appropriate comparison groups, and longer follow-up are needed to clarify the relative role of MOWHTO in this population. Lastly, postoperative outcomes were mainly assessed using KOOS and SF-12 scores. Although these are validated and widely used instruments, they remain patient-reported measures and may be influenced by individual or cultural factors. Future studies incorporating objective functional assessments, such as gait analysis, may provide a more comprehensive evaluation of postoperative recovery.

## Conclusion

MOWHTO was associated with favorable short-term clinical, radiographic, and arthroscopic outcomes in carefully selected patients with medial compartment knee osteoarthritis and mild or moderate varus deformity. No statistically significant differences in postoperative outcomes were detected between the mild- and moderate-varus groups. However, because no nonoperative or alternative surgical comparison group was included, these findings do not establish the superiority or preferential role of MOWHTO over other treatment strategies. These findings are limited to a 2-year follow-up period and should not be interpreted as evidence of long-term disease modification, durable cartilage restoration, or equivalence between the two groups. Longer-term prospective comparative studies are required to determine osteoarthritis progression, osteotomy survivorship, conversion to arthroplasty, and the durability of the observed clinical and arthroscopic changes.

## Data Availability

The original contributions presented in the study are included in the article/Supplementary Material, further inquiries can be directed to the corresponding author.
